# Mucus Sugar Content Shapes the Bacterial Community Structure in Thermally Stressed *Acropora muricata*

**DOI:** 10.3389/fmicb.2016.00371

**Published:** 2016-03-24

**Authors:** Sonny T. M. Lee, Simon K. Davy, Sen-Lin Tang, Paul S. Kench

**Affiliations:** ^1^School of Environment, The University of AucklandAuckland, New Zealand; ^2^School of Biological Sciences, Victoria University of WellingtonWellington, New Zealand; ^3^Microbial Lab, Biodiversity Research Center, Academia SinicaTaipei, Taiwan

**Keywords:** bacteria, *Acropora*, bleaching, pyrosequencing, mucus

## Abstract

It has been proposed that the chemical composition of a coral’s mucus can influence the associated bacterial community. However, information on this topic is rare, and non-existent for corals that are under thermal stress. This study therefore compared the carbohydrate composition of mucus in the coral *Acropora muricata* when subjected to increasing thermal stress from 26 to 31°C, and determined whether this composition correlated with any changes in the bacterial community. Results showed that, at lower temperatures, the main components of mucus were *N*-acetyl glucosamine and C6 sugars, but these constituted a significantly lower proportion of the mucus in thermally stressed corals. The change in the mucus composition coincided with a shift from a γ-*Proteobacteria*- to a *Verrucomicrobiae-* and *α*-*Proteobacteria*-dominated community in the coral mucus. Bacteria in the class *Cyanobacteria* also started to become prominent in the mucus when the coral was thermally stressed. The increase in the relative abundance of the *Verrucomicrobiae* at higher temperature was strongly associated with a change in the proportion of fucose, glucose, and mannose in the mucus. Increase in the relative abundance of α-*Proteobacteria* were associated with GalNAc and glucose, while the drop in relative abundance of γ-*Proteobacteria* at high temperature coincided with changes in fucose and mannose. *Cyanobacteria* were highly associated with arabinose and xylose. Changes in mucus composition and the bacterial community in the mucus layer occurred at 29°C, which were prior to visual signs of coral bleaching at 31°C. A compositional change in the coral mucus, induced by thermal stress could therefore be a key factor leading to a shift in the associated bacterial community. This, in turn, has the potential to impact the physiological function of the coral holobiont.

## Introduction

Corals are known to be associated with a variety of microorganisms, including algae, fungi, bacteria, archaea, and viruses (Knowlton and Rohwer, 2003; Wegley et al., 2007; Marhaver et al., 2008; Lawrence et al., 2014). There is an increasing interest in coral-associated microbes as environmental changes threaten the health of coral reefs, inducing bacterial- (Harvell et al., 1999) and viral- (Lawrence et al., 2014) mediated diseases. The coral holobiont consists of distinct microhabitats – a coral surface mucus layer (SML), tissues, and coral skeleton (Sweet et al., 2011). In particular, the mucus is a carbon-rich compound and serves as an important substrate for bacterial growth (Ferrier-Pagès et al., 2000; Brown and Bythell, 2005). In addition, distinct differences in the bacterial (Cooney et al., 2002; Frias-Lopez et al., 2002) and viral (Lawrence et al., 2014) communities between the SML and water column suggest that water-borne microorganisms do not settle into the layer passively, but rather occupy certain niches within the SML. Various properties of the SML – such as acting as a physical barrier (Cooney et al., 2002), aiding mucociliary food transport to the polyp (Sorokin, 1973; Ducklow, 1990), sloughing of potential pathogenic bacteria (Ducklow and Mitchell, 1979; Rublee et al., 1980), and serving as a medium for anti-bacterial allelochemicals (Slattery et al., 1995, 1997; Koh, 1997; Kelman et al., 1998; Ritchie, 2006) – may prevent pathogenic bacteria from attacking the underlying coral tissues. Rohwer and Kelley (2004) proposed that corals might be able to influence the bacterial community in the SML by altering the composition of the mucus. Therefore, growth of beneficial bacteria and those that inhibit potential pathogens can be promoted for maintaining the wellbeing of the coral host.

In order to penetrate the SML and reach the tissues of a healthy coral host, invading pathogenic bacteria not only have to deal with the coral host defense molecules present in the mucus (Ritchie, 2006), but they must also be able to outcompete members of the native microbiota within the SML (Krediet et al., 2012). For example, native microbiota have been shown to produce extracellular activities that block the induction of enzymes in a white pox pathogen, *Serratia marcescens*, and to prevent the pathogenic bacteria from using the coral mucus as a food source (Krediet et al., 2012). Similarly, even though the known coral pathogens *Vibrio* spp. have been seen to dominate the mucus microcosm under laboratory conditions (Sharon and Rosenberg, 2008; Krediet et al., 2009a), virulence of these bacteria decreases substantially when their ability to efficiently use mucus as a food source is disrupted by either allelochemicals in the coral mucus or the native microbiota’s extracellular activities (Krediet et al., 2012). These observations show that, in order to outcompete the native microbiota within the SML, coral pathogens must have different metabolic capabilities than the native microbes. For instance, even though both pathogens and native microbes produce glycosidases, proteases and esterases to degrade and use coral mucus as a food source (Vacelet and Thomassin, 1991; Krediet et al., 2009a), the regulation, timing, and activity levels of these enzymes are significantly different between bacterial taxa (Sharon and Rosenberg, 2008; Krediet et al., 2009b). Therefore, in order to better characterize the interaction between coral pathogens and native microbiota, it is crucial to understand the relationship between the mucus chemical composition and its microbial communities.

Information about the chemical composition of coral mucus is limited (Meikle et al., 1988; Coffroth, 1990; Vacelet and Thomassin, 1991; Brown and Bythell, 2005; Wild et al., 2005, 2010). Coffroth (1990) described it as a carbohydrate complex, and detailed analysis of *Acropora muricata* mucus revealed that the main component consists of a complex proteoglycan (Meikle et al., 1988). Further analysis of the carbohydrate composition of mucus released by six different *Acropora* species, found arabinose, mannose, galactose, glucose, and *N*-acetyl glucosamine in all samples, and rhamnose, fucose and xylose in some samples (Wild et al., 2005). Besides the coral host, the coral algal symbiont *Symbiodinium* has been shown to synthesize mycosporine-like amino acids, which are then transported to the host (Banaszak and Trench, 1995; Shick et al., 1996; Banaszak et al., 2000). These differences in the carbohydrate composition of the SML highlight the potential for the SML to influence the microbial community composition (Rohwer and Kelley, 2004; Allers et al., 2008). To date, only four studies have tried to link carbohydrate composition of the mucus to bacterial diversity (Ritchie and Smith, 1997, 2004; Klaus et al., 2007; Tremblay et al., 2011). Ritchie and Smith (1997, 2004) isolated and cultured bacteria from the surface of different coral species and showed that these bacteria have specific utilizations of carbon sources. Conversely, a study by Klaus et al. (2007) found no correlation between mucus composition and bacterial diversity in the tissues of *Orbicella annularis*. Finally, Tremblay et al. (2011) found that the bacterial community is species-specific in *Galaxea fascicularis*, *Pavona cactus*, and *Turbinaria reniformis*, and may be linked to the mucus sugar composition and the amount of dissolved organic carbon excreted.

Increasing sea surface temperature can have a negative impact on both the coral host and its microbial associates (Bourne et al., 2009; Mouchka et al., 2010). For example, the pathogenic bacteria *Vibrio* spp. comprised 30% of the entire bacterial community in bleached *O. annularis*, but were absent in healthy coral colonies (Ritchie and Smith, 1997). Similarly, McGrath and Smith (1999) showed that the *Vibrio* spp. population increased during bleaching of *O. annularis*. Unfortunately, no information regarding the effect of thermal bleaching on mucus composition and its subsequent impact on bacterial community composition in the mucus layer is available at present. However, coral bleaching has been shown to induce an increase in organic matter and mucus production (Niggl et al., 2009), while conversely the Caribbean scleractinian coral *Diploria* spp. showed a decrease in its SML thickness when the water temperature was increased to 31°C (Pratte and Richardson, 2014). These studies suggested that there is a possible compositional change to the coral mucus (Wooldridge, 2009). Nevertheless, with contrasting and incomplete results, it is a challenge to understand how the composition of coral mucus influences the associated microbial communities. Furthermore, there is no information on the successive change in mucus composition of thermally stressed corals and their associated surface bacterial communities. This study therefore aimed to address this knowledge gap, by comparing mucus composition and bacterial communities in healthy versus bleached colonies of *A. muricata* over time.

## Materials and Methods

### Mucus Sample Collection

*Acropora muricata* coral nubbins (*n* = 50), approximately 2 cm in length, were collected from five different colonies at 8–10 m depth, from Kenting National Park, Nan-wan, Taiwan (21°57′N, 120°44′E) on May 21, 2013 (permit number Kenting #1002901240). The coral nubbins were acclimated for 30 days in a 0.2-μm filtered seawater (FSW) flow-through tank, with a constant water temperature of 26°C. Light was provided by 400 W HQI metal halide lamps, at an irradiance of ∼150 μmol photons m^-2^ s^-1^ on a 12 h light/dark cycle. After acclimation, 40 nubbins were re-distributed randomly into three treatment tanks and one control tank. All the tanks contained FSW and were illuminated as described above. The control tank was kept at a constant temperature of 26°C. Water temperature in the treatment tanks was raised from 26 to 31°C, at 1°C *per* day. On each sampling day (treatment tank temperatures were 26, 27, 29, and 31°C), one coral nubbin and 1 L FSW were collected from each tank. Coral mucus was then “milked” from each nubbin (Wild et al., 2004) into two 15 mL BD Falcon^TM^ tubes for 5 min each. A total of 16 FSW samples were collected, and 16 replicate coral mucus samples were collected for microbial DNA extraction (*n* = 16) and carbohydrate composition analysis (*n* = 16). Mucus samples from each nubbin were split for microbial DNA analysis and mucus carbohydrate composition analysis. All extracted FSW and coral mucus samples were stored at –20°C until DNA extraction and carbohydrate composition analysis.

A Walz^®^ Diving-pulse amplitude modulated (PAM) fluorometer (0.8 s saturating pulse of >4500 μmol photons m^-2^ s^-1^; gain 12) was used to monitor the change in coral photosystem health. Three dark-adapted yield values *per* nubbin (*F*_v_/*F*_m_) were obtained from three randomly chosen coral nubbins from each tank, an hour after the light was turned off.

### DNA Extraction and Sequencing

Coral mucus was recovered by centrifugation (13,000 × *g* for 15 min), and the final pellet homogenized with 600 μL 10x TE buffer, and incubated with 30 μL 10% SDS and 10 μL 100 μg/mL RNase A for 30 min at 37°C. A 3 μL aliquot of proteinase K (20 mg/mL) was added and incubated at 50°C for 45 min. Finally, after adding100 μL 5 M NaCl and 80 μL CTAB/NaCl solution, the mixture was incubated for another 10 min at 65°C. DNA was then extracted using the chloroform/isoamyl alcohol (24:1), and phenol/chloroform/isoamyl alcohol (25:24:1) method (Sambrook and Russell, 2006). Mucus DNA was precipitated in 0.6x cold 2-propanol, and centrifuged for 8 min at –20°C. The final homogenized solution was transferred to a clean tube and stored at –20°C before 16S rRNA gene PCR amplification. DNA extraction for FSW followed the same procedure as that for coral mucus DNA extraction.

The quantity of extracted DNA was determined using a NanoDrop spectrophotometer (Thermo Scientific, Vantaa, Finland), and standard gel electrophoresis was used to check genomic DNA quality. All samples were PCR amplified using two bacterial universal primers targeting the bacterial V6-V8 hypervariable region of the 16S ribosomal RNA gene (Hamp et al., 2009) with 968F (5′-AAC GCG AAG AAC CTT AC-3′) and 1391R (5′ - ACG GGC GGT GWG TRC-3′). A 30-cycle PCR was conducted under the following conditions: 94°C for 3 min, 94°C for 30 s, 57°C for 10 s, 72°C for 30 s, and 72°C for 2 min as the final extension after the last cycle. The expected DNA band (∼423 bp) was cut from the agarose gel and the DNA was recovered by electroelution (Sambrook and Russell, 2001).

A total of 24 unique tags (16 coral mucus samples, 8 FSW samples) were used in this study, to tag each of the PCR products of the bacterial V6–V8 region from different samples (Chen et al., 2011). The FSW samples for the treatment tanks were pooled for sequencing. Massive parallel pyrosequencing was conducted for pooled 500 ng lots of each tagged V6–V8 DNA sample, using the Roche 454 Genome Sequencer FLX System (Mission Biotech, Taipei).

A total of 63,038 sequences were generated and processed through the mothur software package (Schloss et al., 2009). Sequences of <280 bp length, homopolymer runs exceeding 8 bp, and *q*-scores < 27 (42,950sequences) were removed. Chimerical reads (2,593 sequences) were also removed using UCHIME (Edgar et al., 2011). OTUs were then classified according to their taxonomic affiliations of the V6–V8 sequences (17,495 sequences) using closed-reference OTU picking against Ribosomal Database Project (RDP; v2.10, http://sourceforge.net/projects/rdp-classifier/) with a bootstrap value of 0.8 and 3% cutoff value. An OTU table was compiled at each taxonomic level into a counts file for statistical analysis. Sequences were submitted to the NCBI Sequence Read Archive under accession number SRP060401.

### Carbohydrate Composition

Coral mucus samples were desalted prior to carbohydrate composition analysis using high performance liquid chromatography–mass spectrometry (HPLC–MS). Preparation of the coral mucus followed the procedure outlined in Wild et al. (2010). Briefly, coral mucus samples were desalted using a Spectra/Por Biotech cellulose ester dialysis membrane (molecular weight cutoff of 100–500 Da). Membranes were filled with coral mucus samples and placed in a 4-L container with deionized, sterile water, continuously filling from the bottom and emptying from the top. The whole set-up was placed at 4°C. After 3 days, samples were removed and freeze-dried. Samples were then treated with 1 M HCl in methanol at 80°C for 16 h, after which pyridine and acetic anhydride in methanol were added. Finally, samples were treated with Tri-Sil (Pierce) at 80°C for 0.5 h (York et al., 1986; Merkle and Poppe, 1994) before analysis of carbohydrate composition using HPLC–MS.

The HPLC system (Agilent Infinity 1260 LC system) consisted of a binary pump, an autosampler and a degasser, interfaced to an ESI Turbo V ion source of a QTRAP 5500 (Applied Biosystems, Foster City, CA, USA). The neutral monosaccharides (fucose, xylose, arabinose, *N*-acetyl-galactosamine, *N*-acetyl-glucosamine, mannose, galactose, and glucose) were separated with an Athena NH_2_ column (250 mm × 4.6 mm, 5 μm particle size). Mobile phase A consisted of water and mobile phase B consisted of 100% HPLC-grade acetonitrile (Fisher). A gradient program of 24 to 29% A in 25 min was used, with a 700 μL min^-1^ flow rate and injection volume of 10 μL. The mass spectrometer (Thermo Scientific) was operated in negative ion multiple reaction monitoring (MRM) mode. The [M-H]^-^ precursor ions were used, with the capillary operating at –4500 V, and the source temperature was set to 250°C. The curtain gas (N_2_) and collision gas (N_2_) settings were 20 psi, the nebulization gas setting was 40 psi and the vaporization gas setting was 50 psi. The declustering potential (DP), entrance potential (EP), collision cell exit potential (CXP), and collision energy (CE) were optimized for each analyte.

### Data Analysis

Relative abundances of each bacterial class identified from individual coral mucus samples were used to examine patterns of microbial community structure in the mucus of the healthy and thermally stressed corals. Observed and predicted Chao1 (Chao, 1984) and Simpson (Simpson, 1949) diversity statistics were calculated in MOTHUR and plotted. Data were log transformed and analyzed using multivariate statistical software (PRIMER v6; Clarke, 1993) with PERMANOVA using the Bray–Curtis distance metric to measure the degree of similarity between the bacterial communities in the experiment. The Kruskal–Wallis test and Mann–Whitney *U* with Bonferroni correction for multiple comparisons *post hoc* test were used to determine any significant shifts in the major bacterial communities (γ-*Proteobacteria*, α-*Proteobacteria*, and *Verrucomicrobiae*) of the coral mucus as the water temperature was increased from 26 to 31°C.

Normalized data from mucus carbohydrate analysis were also analyzed using the Kruskal–Wallis test and Mann–Whitney *U post hoc* test (with Bonferroni correction for multiple comparisons), to determine if the mucus composition showed any significant changes between the healthy and thermally stressed corals. Pairwise Wilcoxon test was carried out to determine if there was any significant difference in the coral mucus composition between treatment and control tanks. The relationships between the presence of mucus sugars and the bacterial community composition were ascertained using canonical correspondence analysis (CCA). Additional associations between sugars and the major bacterial taxa (γ-*Proteobacteria*, α-*Proteobacteria* and *Verrucomicrobiae*) were further examined using Spearman’s test. The most closely related genome sequences to the dominant OTUs were then determined by BLAST (Altschul et al., 1997) searches on NCBI websites. The major OTUs potential enzymatic activities on various sugars were obtained from the Kyoto Encyclopedia of Genes and Genomes (KEGG) database (Kanehisa and Goto, 2000). All statistical tests were conducted with the R v3.1.2 statistical programming language with the vegan v2.2-1 package (Oksanen et al., 2015).

## Results

### Thermal Stress

**FIGURE 1:**
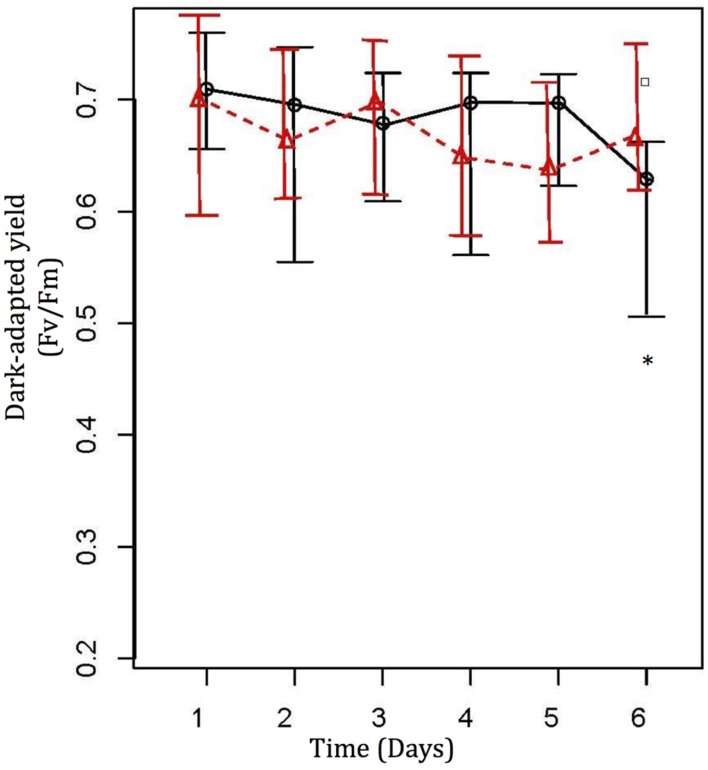
**Photochemical efficiency values (Mean ± SE) from Diving PAM for treatment (O) and control (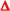) coral nubbins.** Control coral nubbins were kept at a constant 26°C water temperature, whereas temperature in treatment tanks were increased 1°C per day from 26 to 31°C. ^∗^ Represents significant difference at 0.05. Symbol □ represents outliers (Lee et al., 2015).

Water temperature was increased from 26 to 31°C, at a rate of 1°C *per* day. Maximum photochemical quantum yield (*F*_v_/*F*_m_) of *A. muricata* in the treatment tank fell from 0.705 ± 0.03 at 26°C to 0.604 ± 0.072, with an outlier at 0.719 (not removed) at 31°C (**Figure 1**; one-way ANOVA, *F*_5,15_ = 8.976, *p* < 0.001), while values in the control tank remained consistent at 0.693 ± 0.057 (**Figure 1**). *Post hoc* analysis revealed that the most significant thermal impact on the corals was at 31°C (*p* < 0.001), which was consistent with the visual assessment of bleaching. Comparing changes in photochemical quantum yield (*F*_v_/*F*_m_) of *A. muricata* between the treatment and control tanks revealed significant thermal impact at 31°C (**Figure 1**; one-way ANOVA, *F*_11,318_ = 4.444, *p* < 0.001; *post hoc* analysis – *p* = 0.001).

### Mucus Sugar Composition

**FIGURE 2:**
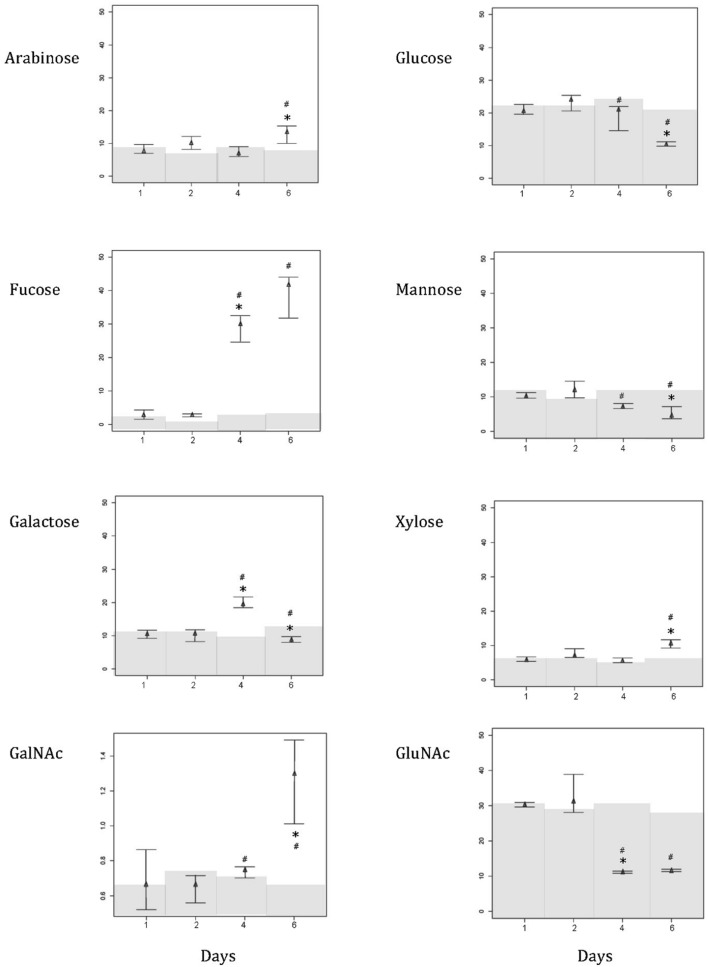
**Composition shift (in percentage) of various sugar in the mucus layer of *Acropora muricata* from day 1 to day 6.** Water temperature started at 26°C on day 1, and was increased 1°C from 26 to 31°C. Temperature in control tank was constant at 26°C. Note that the scale for GalNAc is different from the rest of the sugars in order to show the changing trend. Gray regions represent the relative composition of the various sugar in the control samples. ^∗^ Represents significant difference between treatment tanks, and ^#^ represents significant difference between control and treatment tanks at 0.05.

At 26°C, mucus was mostly composed of glucose (20.855 ± 1.56%), mannose (10.408 ± 0.799%), galactose (10.519 ± 1.235%), and *N*-acetyl glucosamine (GluNAc, 30.212 ± 0.639%). Arabinose (7.998 ± 1.476%), fucose (2.861 ± 1.386%), and xylose (6.088 ± 0.659) contributed less, and *N*-acetyl galactosamine was barely noticeable (GalNAc, 0.683 ± 0.172%; **Figure 2**). As the temperature increased to 31°C, all sugars experienced a significant shift (decrease or increase) in their relative proportions [**Figure 2**; Kruskal–Wallis chi-squared: arabinose, *H*(3) = 7.732, *p* = 0.050; fucose, *H*(3) = 8.949, *p* = 0.029; galactose, *H*(3) = 7.615, *p* = 0.050; GalNAc, *H*(3) = 7.308, *p* = 0.049; glucose, *H*(3) = 7.615, *p* = 0.050; mannose, *H*(3) = 8.949, *p* = 0.029; xylose, *H*(3) = 9.154, *p* = 0.027; GluNAc, *H*(3) = 8.744, *p* = 0.033]. Significant shifts in the proportions of mucus sugars occurred at 29 and 31°C (**Figure 2**). However, not all sugars exhibited significant changes in their relative proportions at the same temperature. For example, at 29°C, fucose showed a significant increase (*p* < 0.001, 29.039 ± 4.043), while GluNAc showed a significant decrease (*p* = 0.003, 11.117 ± 0.324) in their relative contributions. At a higher temperature (31°C), there was an increase in the proportion of arabinose (*p* = 0.041, 12.958 ± 2.704), GalNAc (*p* = 0.008, 1.268 ± 0.243), and xylose (*p* = 0.005, 10.639 ± 1.231), but the relative proportions of glucose (*p* = 0.005, 10.504 ± 0.508) and mannose (*p* = 0.032, 5.09 ± 1.865) decreased (**Figure 2**). The proportion of galactose increased significantly (*p* < 0.001, 19.604 ± 1.832) at 29°C, but dropped acutely at 31°C (*p* < 0.001, 8.893 ± 0.873). There were no significant changes in the relative proportions of sugars in the control tank [**Figure 2**; Kruskal–Wallis chi-squared: arabinose, *H*(3) = 1.257, *p* = 0.739; fucose, *H*(3) = 4.593, *p* = 0.204; galactose, *H*(3) = 3.359, *p* = 0.339; GalNAc, *H*(3) = 2.895, *p* = 0.408; glucose, *H*(3) = 3.205, *p* = 0.361; mannose, *H*(3) = 3.102, *p* = 0.376; xylose, *H*(3) = 5.974, *p* = 0.113; GluNAc, *H*(3) = 1.769, *p* = 0.622]. Comparing the coral mucus samples from the treatment tanks and the control tank, there is a significant difference in the relative proportion of xylose [**Figure 2**; Kruskal–Willis chi-squared; *H*(3) = 10.697, *p* = 0.013; *post hoc* analysis – *p* < 0.001] and arabinose [**Figure 2**; *H*(3) = 7.401, *p* = 0.049; *post hoc* analysis – *p* < 0.001] at 31°C. Other sugars exhibited significant differences at both 29 and 31°C [**Figure 2**; fucose: *H*(3) = 11.272, *p* = 0.010; *post hoc* analysis – *p* = 0.014 (29°C), *p* = 0.032 (31°C); galactose: *H*(3) = 9.284, *p* = 0.026; *post hoc* analysis – *p* = 0.005 (29°C), *p* = 0.006 (31°C); GalNAc: *H*(3) = 9.599, *p* = 0.022; *post hoc* analysis – *p* = 0.005 (29°C), *p* = 0.005 (31°C); glucose: *H*(3) = 9.320, *p* = 0.025; *post hoc* analysis – *p* = 0.006 (29°C), *p* = 0.005 (31°C); mannose: *H*(3) = 11.424, *p* = 0.009; *post hoc* analysis – *p* = 0.006 (29°C), *p* = 0.006 (31°C); GluNAc: *H*(3) = 11.214, *p* = 0.011; *post hoc* analysis – *p* = 0.003 (29°C), *p* = 0.003 (31°C)].

### Bacterial Community Changes

**FIGURE 3:**
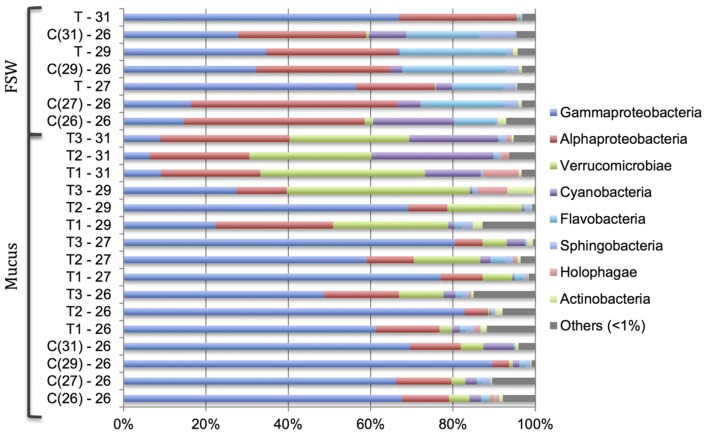
**Changes in relative abundance of bacterial community at the class level in the *Acropora muricata* coral mucus (Mucus) and filtered seawater (FSW) from 26 to 31°C.** Control tanks (C) and treatment tanks (T) and numbers represents the respective temperatures. Numbers in the ( ) in the control samples (C) represent the respective treatment temperature. For example, C(31) – 26 represents control sample at 26°C when the temperature in the treatment tanks were 31°C.

There was a significant difference in the bacterial community composition in the coral SML between samples from the different treatments (26–31°C; PERMANOVA, Pseudo-*F*_3,15_ = 10.166, *P* = 0.001; **Figure 3**). However, there was no significant change in bacterial community structure in the control tanks that were maintained at 26°C (PERMANOVA, Pseudo-*F*_3,6_ = 0.899, *P* = 0.644; **Figure 3**). Members of the class γ-*Proteobacteria* dominated the bacterial community in the coral mucus at the lower temperatures (26–29°C). However, at 31°C, members from the class α-*Proteobacteria* and *Verrucomicrobiae* became more prominent (**Figure 3**). As the temperature increased from 26 to 31°C, the relative abundance of α-*Proteobacteria* and *Verrucomicrobiae* increased from 12.57 ± 2.84% to 27.09 ± 1.60%, and 4.49 ± 2.46% to 33.82 ± 4.09%, respectively. On the other hand, relative abundances of γ-*Proteobacteria* dropped markedly, from 62.20 ± 8.70% to 8.26 ± 0.88%. Changes in relative abundances of α-*Proteobacteria*, *Verrucomicrobiae* and γ-*Proteobacteria* were significant (Kruskal–Wallis chi-squared; α-*Proteobacteria*, *H*(3) = 7.821, *p* = 0.049; *Verrucomicrobiae*, *H*(3) = 8.949, *p* = 0.029; γ-*Proteobacteria*, *H*(3) = 8.689, *p* = 0.016). *Post hoc* analysis revealed that the most significant shifts in the bacterial community occurred at 31°C (α-*Proteobacteria*, *p* = 0.011; *Verrucomicrobiae*, *p* = 0.018; γ-*Proteobacteria*, *p* = 0.031). There was no significant change in the bacterial community composition in the control tank. In FSW, the bacterial community was dominated by α-*Proteobacteria*, but there was a prominent increase in the relative abundance of γ-*Proteobacteria* at 31°C (**Figure 3**).

**FIGURE 4:**
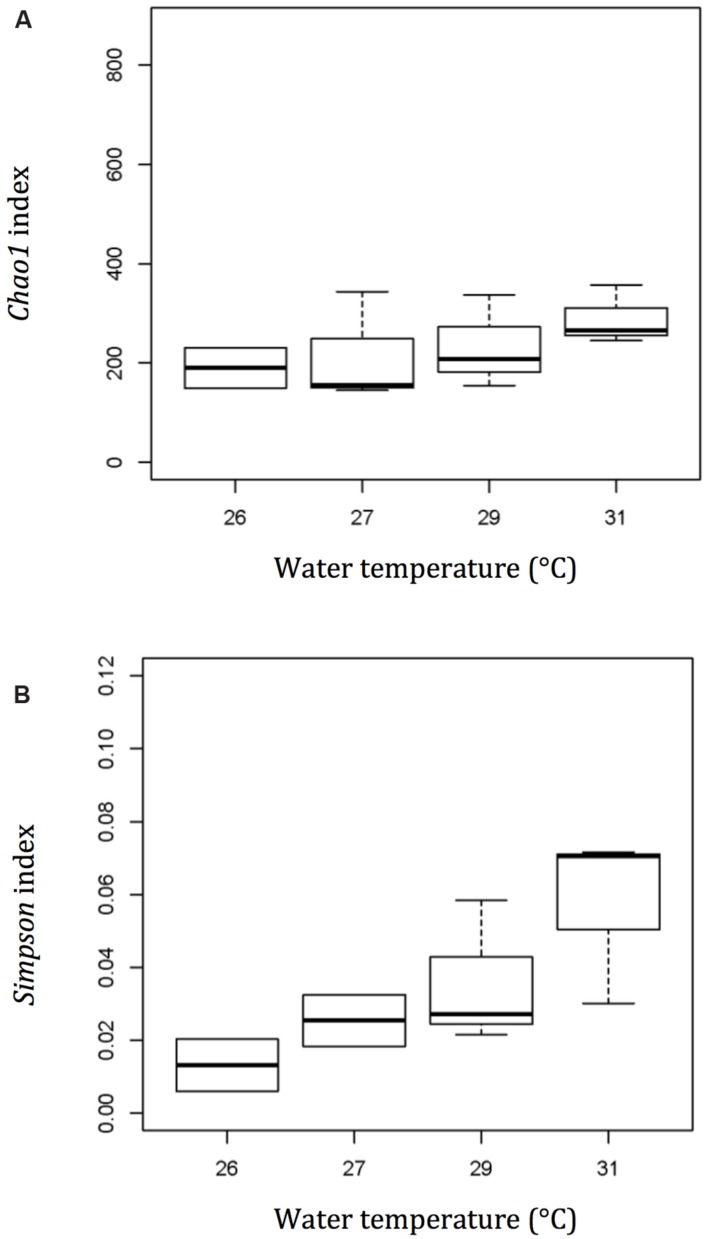
**Diversity and richness index – *Chao1* (A) and Simpson (B) show a more diverse bacterial community in the mucus layer of thermally stressed *Acropora muricata* at a higher temperature.** Plot – line represents median values, boxes represent 75% percentile and whiskers represent the highest and lowest values.

As the temperature increased from 26 to 31°C, both the Chao1 and Simpson diversity indices increased steadily (**Figure 4**). The Chao1 index increased from 193.50 ± 57.63 at 26°C to 289.01 ± 59.75 at 31°C, while the Simpson diversity index increased from 0.013 ± 0.010 to 0.057 ± 0.024.

### Relationship between Bacterial Community and Mucus Composition

The major bacterial OTUs that were retrieved from the coral mucus samples, and were affiliated with bacterial genomes from the NCBI database, belonged to the classes γ-*Proteobacteria*, *Verrucomicrobiae*, and *Cyanobacteria* (**Table 1**). Based on KEGG pathway annotations on the most closely related genome sequences, most of the OTUs showed potential enzymatic activities on at least two of the mucus sugars tested. Bacterial OTUs affiliated to *Verrucomicrobiae* (*Rubritalea halochordaticola* and *Rubritalea sabuli*) showed potential enzymatic activities on GalNAc, glucose, and GluNAc. OTUs affiliated to *Prochlorococcus marinus* (*Cyanobacteria*) potentially use galactose, glucose, and GluNAc. Out of the eight tested sugars, identified OTUs that were affiliated to *Nostoc punctiforme* (*Cyanobacteria)* can potentially utilize glucose. All identified OTUs that were affiliated to γ-*Proteobacteria* potentially can utilize glucose, but *Vibrio coralliilyticus* (arabinose, fucose, galactose, GalNAc, and glucose) and *Vibrio natriegens* (galactose, GalNAc, glucose, GluNAc, mannose, and xylose) affiliated OTUs may potentially be able to utilize a wide range of sugars as their food source (**Table 1**).

**Table 1:** **(A)** Affiliation of bacterial sequences retrieved from coral mucus samples.

OTU	Composition	Class	Closest relative	Database accession number	Similarity (%)
OTU0001	12.61%	*Verrucomicrobiae*	*Rubritalea halochordaticola* strain MN1-1006	NR_113049.1	100%
OTU0002	7%	γ*-Proteobacteria*	*Endozoicomonas montiporae* strain CL-33	NR_116609.1	96%
OTU0023	6%	γ*-Proteobacteria*	*Vibrio coralliilyticus* ATCC BAA-450	NR_117892.1	99%
OTU0003	4.88%	*Verrucomicrobiae*	*Rubritalea sabuli* strain YM29-052	NR_041630.1|	97%
OTU0016	4%	*Cyanobacteria*	*Prochlorococcus marinus* ssp. *pastoris* str. PCC 9511	NR_028762.1	90%
OTU0015	4.31%	γ*-Proteobacteria*	*Vibrio natriegens* strain NBRC 15636	NR_113786.1|	99%
OTU0005	3.13%	γ*-Proteobacteria*	*Endozoicomonas elysicola* strain MKT110	NR_041264.1	96%
OTU0009	2.49%	*Cyanobacteria*	*Nostoc punctiforme* strain PCC 73102	NR_074317.1|	92%

**Table 1:** **(B)** Affiliation of bacterial sequences retrieved from coral mucus samples and their potential enzymatic activities on various sugar.

OTU	Arabinose	Fucose	Galactose	GalNAc	Glucose	GluNAc	Mannose	Xylose
OTU0001				+	+	+		
OTU0002			+		+			
OTU0023	+	+	+	+	+		+	
OTU0003				+	+	+		
OTU0016			+		+	+		
OTU0015			+	+	+	+	+	+
OTU0005		+	+		+	+	+	+
OTU0009					+			

*Only top OTUs which have a mean relative abundance of more than 1% of the total bacterial community are shown. Note: Genomes from **Table 1A** were used for inference of potential enzymatic activities*.

**FIGURE 5:**
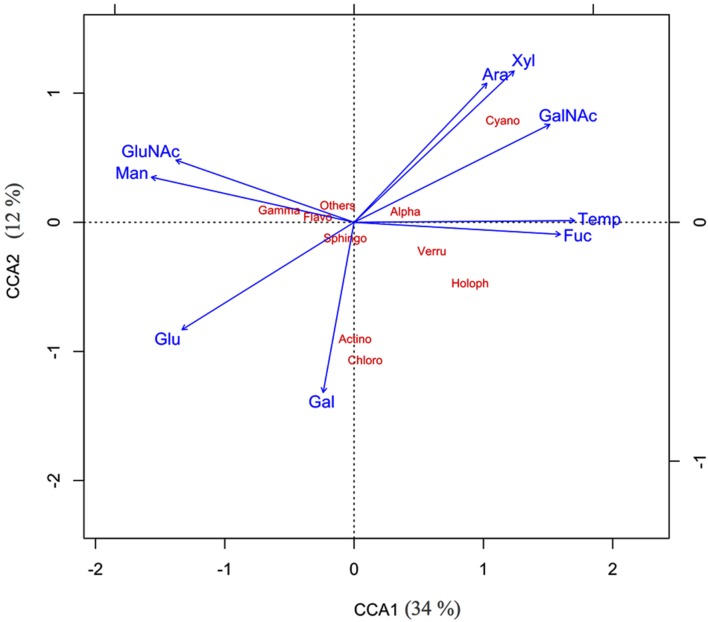
**Canonical component analysis – CCA1 (34%), CCA2 (12%) showed the relationship between various mucus sugar and major bacterial classes of thermally stressed *Acropora muricata***.

The two CCA axes of the ordination had eigenvalues of 0.34 and 0.12, respectively, showing that the combined pool of mucus sugars accounted for about 46% of the total variation in the bacterial community composition in the coral mucus (**Figure 5**). The bi-plot revealed an apparent segregation between a group of sugars that decreased with increasing temperature (glucose, mannose, and GluNAc) and the other group of sugars that increased at higher temperature (arabinose, xylose, fucose, and GalNAc).

Spearman’s correlation tests revealed that the relative abundance of α-*Proteobacteria* was correlated with the presence of GalNAc (*p* = 0.019, *r* = 0.629) and glucose (*p* = 0.051, *r* = –0.580). Three additional key relationships were noted: (1) The relative abundance of *Verrucomicrobiae* was strongly associated with the presence of fucose (*p* = 0.005, *r* = 0.776), glucose (*p* = 0.017, *r* = –0.685) and mannose (*p* < 0.001, *r* = –0.881); (2) the relative abundance of γ-*Proteobacteria* was associated with fucose (*p* = 0.04, *r* = –0.608) and mannose (*p* = 0.032, *r* = 0.629); and (3) the presence of *Cyanobacteria* was highly associated with arabinose (*p* = 0.001, *r* = 0.819) and xylose (*p* = 0.002, *r* = 0.811). Other identified sugars had no statistical effects on the bacterial community of the SML.

## Discussion

This study showed that the bacterial community associated with the mucus of the coral *A. muricata* becomes more diverse as the coral host experiences thermal stress. Furthermore, the bacterial community shifts from being dominated by γ-*Proteobacteria* to α-*Proteobacteria* and *Verrucomicrobiae* at higher temperatures, while *Cyanobacteria* also start to appear in the mucus as the coral becomes thermally stressed. The shift in the bacterial community could be linked to a compositional change of the exuded mucus.

### Mucus Composition and Its Impact on Bacterial Diversity

Thermal stress and bleaching have an impact on mucus composition (Wooldridge, 2009), suggesting that this could in turn influence the microbial community. This was apparent in our study, where *A. muricata* showed signs of bleaching both visually and photochemically at 31°C, accompanied by an increase in the relative proportions of GalNAc and fucose in the mucus, and an increase in the relative abundance of *Rubritalea halochordaticola* (*Verrucomicrobiae*) and *Vibrio coralliilyticus* (γ-*Proteobacteria*) affiliated sequences. Both of these sugars are known to be utilizable by these bacterial species (Ben-Haim et al., 2003; Yoon et al., 2011), while the increase in *Vibrio* spp. seen here is consistent with that observed previously in bleached and diseased corals (Mouchka et al., 2010). The dominant OTUs were most closely related to the genome sequences affiliated with *R. halochordaticola* and *R. sabuli* (*Verrucomicrobiae*), which do not harbor annotated KEGG pathways for metabolism of fucose. However, a previous study (Yoon et al., 2011) indicated the usage of fucose by a different strain of *R. halochordaticola*. Although the reason for this contradiction cannot be determined here, the high level of genomic diversity among even closely related bacteria makes it probable that at a lower taxonomic level, retrieved sequences that were affiliated to *R. halochordaticola* and *R. sabuli* do not have the ability to utilize fucose as their food source. Further culturing experiments with *R. halochordaticola, R. sabuli*, and other bacteria of the class *Verrucomicrobiae* will provide insights into the ability of these microbes to use different sugars. Bacterial communities belonging to the γ-*Proteobacteria* (sequences affiliated to *Endozoicomonas montiporae, E. elysicola, V. coraliilyticus*, and *V. natriegens*) can utilize a wide variety of sugars tested in this study. With the exception of *E. elysicola*, both of the retrieved *Vibrio* spp. sequences have the ability to utilize six out of the eight sugars. At higher seawater temperatures, there was a change in coral mucus composition, resulting in a decrease in certain sugars (such as glucose, galactose, mannose, and GluNAc). It is plausible that the ability to use a wide variety of sugars enables *Vibrio* spp. to outcompete the native microbiota and proliferate in the coral SML at higher temperatures (Krediet et al., 2012). Hence, we speculate that the change in mucus composition of thermally stressed *A. muricata* favors the growth of certain bacteria, such as *Vibrio* spp., resulting in the proliferation of these bacteria in the SML.

Coral mucus contains high concentrations of polysaccharides that favor bacterial growth (Ferrier-Pagès et al., 2000; Wild et al., 2004), and the sugars identified in our study are common in the mucus of many corals (Ducklow and Mitchell, 1979; Meikle et al., 1988; Wild et al., 2005). Given this compositional diversity, changes in the sugar content have the potential to influence microbial populations in the coral holobiont (Benson and Muscatine, 1974; Ducklow and Mitchell, 1979; Wild et al., 2004). It should be noted though, that other, interrelated factors could play a role too. In particular, given that coral-associated bacterial communities have as many as 6000 unique ribotypes (Rohwer et al., 2002), spatial and temporal heterogeneity could arise as a result of complex interactions between members of the diverse bacterial community (Torsvik et al., 2002), while physical factors such as mucus structure (Ducklow and Mitchell, 1979) and the amount of mucus exuded (Fitt et al., 2009; Piggot et al., 2009) could also cause changes in the bacterial community. Furthermore, in this study, although visual evidence of bleaching at 31°C corresponded to significant changes in both mucus composition and the bacterial community, a shift in the SML bacterial community could be an indirect consequence of stress to the symbiotic *Symbiodinium* cells, which might have an impact on the production of sugars and other factors that influence microbial community structure (Ritchie, 2006; DeSalvo et al., 2010; Rosic et al., 2011; Rocker et al., 2012). It was not possible to conclude here whether or not a shift in the SML microbiota was a direct result of changes to the mucus composition, but our data suggest that mucus sugars could potentially shape the SML microbial community structure. The influence of other factors on mucus composition and the resulting microbial community composition requires further investigation.

### Implications of Changes in Bacterial Diversity for Coral Health

Changes in coral health can cause a shift in the bacterial community, which may in turn result in further physiological deterioration of the coral (Bourne et al., 2008). In our study, the native microbiota within the coral SML was dominated by members of the class γ-*Proteobacteria*. There were very few changes in the bacterial community composition in the control samples, as well as samples at the lower seawater temperature. However, at higher temperatures, not only was the bacterial diversity higher, but the bacterial community shifted to being dominated by members of the α-*Proteobacteria* and *Verrucomicrobiae*, with *Cyanobacteria* becoming more prominent as well. It is plausible that potentially opportunistic pathogens from the α-*Proteobacteria*, *Verrucomicrobiae*, and *Cyanobacteria* are more efficient at utilizing the mucus of the coral host at the higher temperatures, thus enabling them to overgrow and replace members of the γ-*Proteobacteria*, and dominate the coral-associated bacterial community in the SML. It is hypothesized that the coral SML acts as a first defense of the coral against any invading pathogens (Brown and Bythell, 2005). The coral SML can act as a physical barrier to the surrounding seawater microbes (Cooney et al., 2002), as well as serve as a medium in which anti-bacterial allelochemicals may be exuded (Slattery et al., 1995, 1997; Koh, 1997; Kelman et al., 1998; Ritchie, 2006). Furthermore, potential pathogens must be able to outcompete members of the native microbiota within the SML before they can invade the coral’s tissues (Krediet et al., 2012).

Similarly, in the coral mucus, even though the native microbiota (commensals and mutualists) and pathogens within the SML produce similar exoenzymes to utilize coral mucus, there is a difference in their levels of activity and regulation (Sharon and Rosenberg, 2008; Krediet et al., 2009b). In thermally stressed corals, although both native microbiota and potential pathogenic members of the α-*Proteobacteria*, *Verrucomicrobiae*, and *Cyanobacteria* could show strong catabolite repression by sugars present in coral mucus and adapt to the existing and preferred sugar in the coral SML, differences in temporal regulation of exoenzymes enable potential pathogens to rapidly proliferate (Krediet et al., 2009b). Commensal and/or mutualistic bacteria within the SML might not be able to regulate their glycosidases efficiently at high temperature for the sugars present (Krediet et al., 2009b). This would allow potential pathogens to proliferate and outcompete the native microbiota within the mucus. In a study by Krediet et al. (2009a), a white pox pathogen, *Serratia marcescens* PDL100, showed strong catabolite repression by the sugars present in coral mucus in the early stages of mucus colonization, indicating that the coral pathogen used constitutively active glycosidases to outcompete commensals in the SML. As a result, the native microbiota was not able to produce extracellular activities such as antibiotic production, inhibition of quorum sensing and secondary metabolite production (Shnit-Orland and Kushmaro, 2009; Teplitski and Ritchie, 2009; Rypien et al., 2010) to block the induction of glycosidases in the pathogens, and was thus unable to interfere with the ability of the pathogenic bacteria to use the coral mucus (Krediet et al., 2012).

## Conclusion

The interaction between the coral host, coral-associated commensal and mutualistic bacteria, and invading pathogens is highly complex and still poorly understood. Results from our study demonstrate that thermally stressed corals have a different mucus composition than healthy corals, which may have an impact on the associated bacterial community in the SML. Although the method used in this study to extract the mucus from the coral samples (‘milked’ mucus) may have underestimated the bacterial diversity in the SML when compared to other techniques, such as the novel ‘snot-sucker’ (Sweet et al., 2011), changes to the relative abundance of potential coral-associated pathogens provided an insight into the functions of the coral mucus. The increase in relative abundance of pathogenic bacteria such as *Vibrio* spp. in the SML suggests that these bacteria may have the ability to efficiently regulate certain enzymatic activities to utilize the mucus more efficiently (Krediet et al., 2009b) as the mucus composition changes under thermal stress. These pathogenic bacteria could potentially reduce the expression of enzymes for other sugars and utilize the best monosaccharide available in the coral mucus as their primary carbon source. The shift in the proportion of different types of sugars in the SML, coinciding with a change in the bacterial community, suggests that, as nutrients become less available when the coral’s health is being compromised, potential pathogens may become more efficient in utilizing the different sugars (Teplitski et al., 2006; Deutscher, 2008; Gorke and Stulke, 2008). Therefore, it is highly plausible that the different strategies adopted by the native microbiota and pathogens to colonize the SML would allow opportunistic pathogens an advantage when there is a change in mucus composition in thermally stressed corals. Further research will elucidate the differences in the strategies employed by coral commensals, mutualists and opportunistic pathogens, and may help define their functions within the coral SML.

## Author Contributions

SL: experimental design and execution, data analysis, and writing the manuscript. SD, ST, and PK: writing the manuscript.

## Conflict of Interest Statement

The authors declare that the research was conducted in the absence of any commercial or financial relationships that could be construed as a potential conflict of interest.
